# High Uric Acid (UA) Negatively Affects Serum Tartrate-Resistant Acid Phosphatase 5b (TRACP 5b) Immunoassay

**DOI:** 10.1371/journal.pone.0147554

**Published:** 2016-01-22

**Authors:** Zhi-Qi Wu, Yan Zhang, Erfu Xie, Wei-Juan Song, Rui-Xia Yang, Cheng-Jing Yan, Bing-Feng Zhang, Hua-Guo Xu

**Affiliations:** Department of Laboratory Medicine, the First Affiliated Hospital of Nanjing Medical University, Nanjing, Jiangsu Province, China; Florida State University, UNITED STATES

## Abstract

**Background:**

Bone metastases often occur in the majority of patients with advanced cancer, such as prostate cancer, lung cancer and breast cancer. Serum tartrate-resistant acid phosphatase 5b (TRACP 5b), a novel bone resorption marker, has been used gradually in the clinics as a specific and sensitive marker of bone resorption for the early diagnosis of cancer patients with bone metastasis. Here, we reported that high concentrations of uric acid (UA) lead to decrease of TRACP 5b levels and determined whether TRACP 5b level was associated with UA in interference experiment.

**Methods:**

A total of 77 patients with high concentrations of UA and 77 healthy subjects were tested to evaluate the differences in their TRACP 5b levels. Serial dilutions of UA were respectively spiked with a known concentration of TRACP 5b standard sample, then Serum TRACP 5b was detected by using bone TRAP^®^ Assay. A correction equation was set to eliminate UA-derived TRACP 5b false-decrease. The effect of this correction was evaluated in high-UA individuals.

**Results:**

The average TRACP level of the high-UA individuals (1.47± 0.62 U/L) was significantly lower than that of the healthy subjects (2.62 ± 0.63 U/L) (t-test, p<0.0001). The UA correction equation derived: ΔTRACP 5b = -1.9751lgΔUA + 3.7365 with an R^2^ = 0.98899. Application of the UA correction equation resulted in a statistically non-significant difference in TRACP 5b values between the healthy subjects and high-UA individuals (p = 0.24).

**Conclusions:**

High UA concentrations can falsely decrease TRACP 5b levels due to a method-related systematic error. To avoid misdiagnoses or inappropriate therapeutic decisions, increased attention should be paid to UA interference, when TRACP 5b is used for early diagnosis of cancer patients with bone metastasis, evaluation of the aggressiveness of osteosarcoma or prediction of survival in prostate cancer and breast cancer with bone metastases.

## Introduction

With very few exceptions, the natural history of all kinds of tumors is known to progress from localized indolent stages to aggressive metastatic stages[1,2]. Once metastasis occurs, most patients become incurable[3]. Bone metastases are common in tumor metastases. Bone metastases often occur in the majority of patients with advanced cancer, such as prostate cancer[4], lung cancer[5] and breast cancer[6]. Bone metastases can cause severe pain to the patients, as well as significant resource requirements and costs to the care providers. Bone metastases can also lead to significant morbidity such as bone pain, pathological fractures, impaired mobility, hypercalcemia and spinal cord compression[7–10]. Accurate reliable detection of metastatic bone disease is very important for primary staging because it could affect the therapeutic decision[7]. The diagnosis of bone metastasis is usually performed initially with bone scintigraphy screening and confirmed by plain radiography and/or computed tomography or magnetic resonance imaging[11]. Although the sensitivity of bone scintigraphy is quite high, its specificity is not satisfactory because of false-positive values caused by inflammation and traumatic fracture[12].

Tartrate-resistant acid phosphatase 5b (TRACP 5b) is generally secreted by osteoclasts during bone resorption[13,14]. Its activity can be specifically measured in serum by immunoassays and has been devised as a marker of bone resorption[5,6,15,16]. The role of serum TRACP 5b has been well documented in diseases with a high bone resorption rate, such as osteoporosis, multiple myeloma, bone metastases from breast cancer, lung cancer, and prostate cancer[17–20]. Serum TRACP 5b has been used gradually in the clinics as a specific and sensitive marker of bone resorption for the diagnosis of cancer patients with bone metastasis [21,22], for the evaluation of the aggressiveness of osteosarcoma [23], and as a marker of late loosening of total hip arthroplasty [24]. In addition, TRACP 5b has been proved to be predictive of survival in prostate cancer and breast cancer with bone metastases[25,26].

Uric acid (UA) is a heterocyclic compound of carbon, nitrogen, oxygen, and hydrogen with the formula C5H4N4O3. UA is a product of the metabolic breakdown of purine nucleotides. High blood concentrations of UA can lead to gout[27]. High intake of dietary purine, high-fructose corn syrup, table sugar, and certain drugs such as thiazide diuretics can cause increased levels of UA. Hyperuricemia is associated with increased risk of colorectal, breast, prostate, and other cancers[28–30]. Meanwhile, cancer itself could promote hyperuricemia through cancer related cell death, due to cancer or cancer treatments[31].

Recently, we observed that sixteen cancer patients with bone metastasis diagnosed by bone scintigraphy showed no obviously increased TRACP 5b levels. Meanwhile, these patients showed higher UA level than others. Here, we investigated the correlation between the concentrations of UA and TRACP 5b in a random sample of 77 high-UA patients and determined whether TRACP 5b level was associated with UA in interference experiments.

## Materials and Methods

### Serum sampling

This study was approved by the Ethics Committee of the First Affiliated Hospital of Nanjing Medical University. All samples were collected from August 2014 to June 2015. Patients with cancer, hepatitis, renal dysfunction and inflammatory disease were excluded from serum collection. A total of 77 patients (Hyperuricemia), including 16 women and 61 men (median age, 36 yr; range, 17–66 yr) formed the study group. Additionally, 77 healthy subjects were sampled as the control group, including 16 women and 61 men (median age, 43 yr; range, 22–66 yr).

### Data collection

The alanine aminotransferase (ALT), aspartate aminotransferase (AST), blood glucose (Glu), blood urea nitrogen (BUN), creatinine (CREA) and uric acid (UA) quantitation were analyzed using an Olympus AU5400 automatic chemical analyzer and commercial kits (Olympus, Janpan) according to the instruction manual. White blood cells (WBC) and neutrophils (NEU) % were counted by the Sysmex XE-2100 hematology analyzer (Sysmex, Kobe, Japan). The levels of carcinoembryonic antigen (CEA) and alpha-fetoprotein (AFP) were measured by electrochemiluminescence immunoassay (ECLIA) on an Elecsys E-602 (Roche Diagnostics, Basel, Switzerland). Serum tartrate-resistant acid (TRACP) 5b was detected by using bone TRAP^®^ Assay (IDS Ltd, Boldon, UK).

### UA interference experiment and derivation of a UA correction equation

A known concentration of TRACP 5b standard sample was divided into 6 aliquots. Serial dilutions of UA (250, 500, 1000, 2000, 4000μM) were prepared from UA standard subject. The 5 aliquots were then spiked at 3:2 with each UA solution. This generated 5 different test samples with the same TRACP 5b level, whose final UA concentrations ranged from 100 to 1600 μM. An aliquot containing DDW (double distilled water) instead of UA served as a blank. Serum TRACP 5b assay was performed according to manufacturer’s protocol. The change in TRACP 5b level caused by UA spikes was measured and marked as a function of UA. A linear formulation for the effect of UA-derived TRACP 5b was made based on the best least squares fit.

### Performance evaluation of the correction equation

The concentrations of both TRACP 5b and UA were measured in serum sample of each donor. Each high-UA individual's UA-corrected TRACP 5b concentration was calculated based on the UA-TRACP 5b correction equation. Increasing UA concentration means difference value between each high-UA individual's and mean value of healthy subjects’.

### Statistical analysis

Results were presented as means and ranges. Statistical analysis was performed with SPSS 16.0. Two-tailed t-tests were used for significance testing between groups of continuous data. Corrected TRACP 5b concentration was calculated according to the formula: ΔTRACP 5b (U/L) = -1.9751lgΔUA (μM) + 3.7365. For all statistical comparisons a p< 0.05 was considered statistically significant.

### Ethical standards and patient consent

Ethical clearance for this study was obtained from the Ethics Committee at the First Affiliated Hospital of Nanjing Medical University. Because all the samples used in this study were collected from clinical residual specimen, written informed content from each patient was waived.

## Results and Discussion

**Fig 1:**
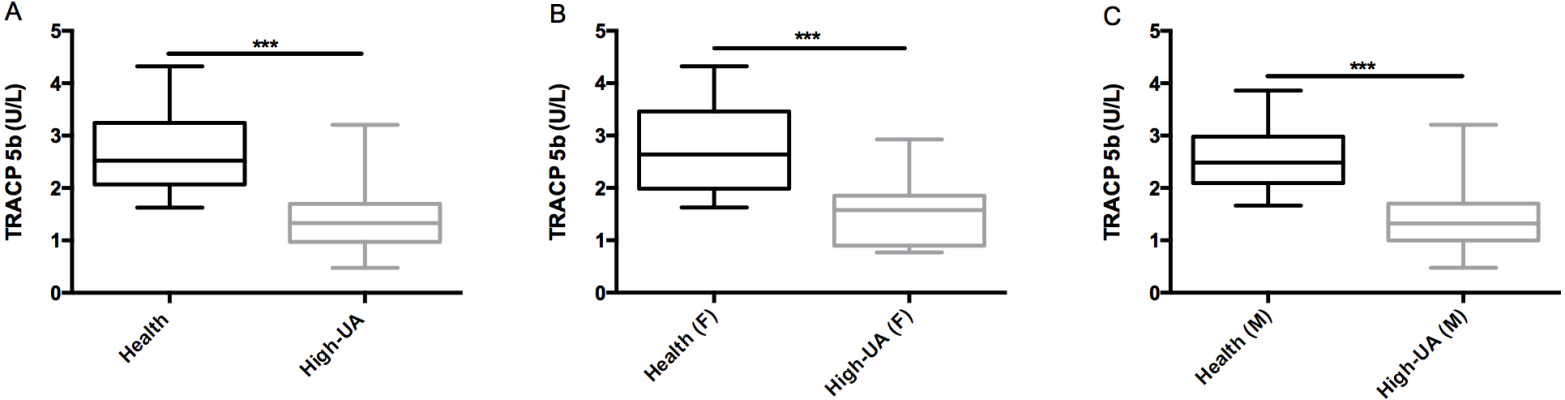
Negative correlation between the concentrations of UA and TRACP 5b. (A) The average TRACP 5b levels of the 77 high-UA individuals (1.47± 0.62 U/L) were significantly lower (t-test, p<0.0001) than that of the 77 healthy subjects (2.62 ± 0.63U/L). (B) The average TRACP 5b levels of the 16 female high-UA individuals (1.51 ± 0.64 U/L) were significantly lower (t-test, p<0.0001) than that of the 16 female healthy subjects (2.73 ± 0.86 U/L). (C) The average TRACP 5b levels of the 61 male high-UA individuals (1.46 ± 0.62 U/L) were significantly lower (t-test, p<0.0001) than that of the 61 male healthy subjects (2.59 ± 0.56 U/L). Triple asterisk indicates p<0.0001.

**Fig 2:**
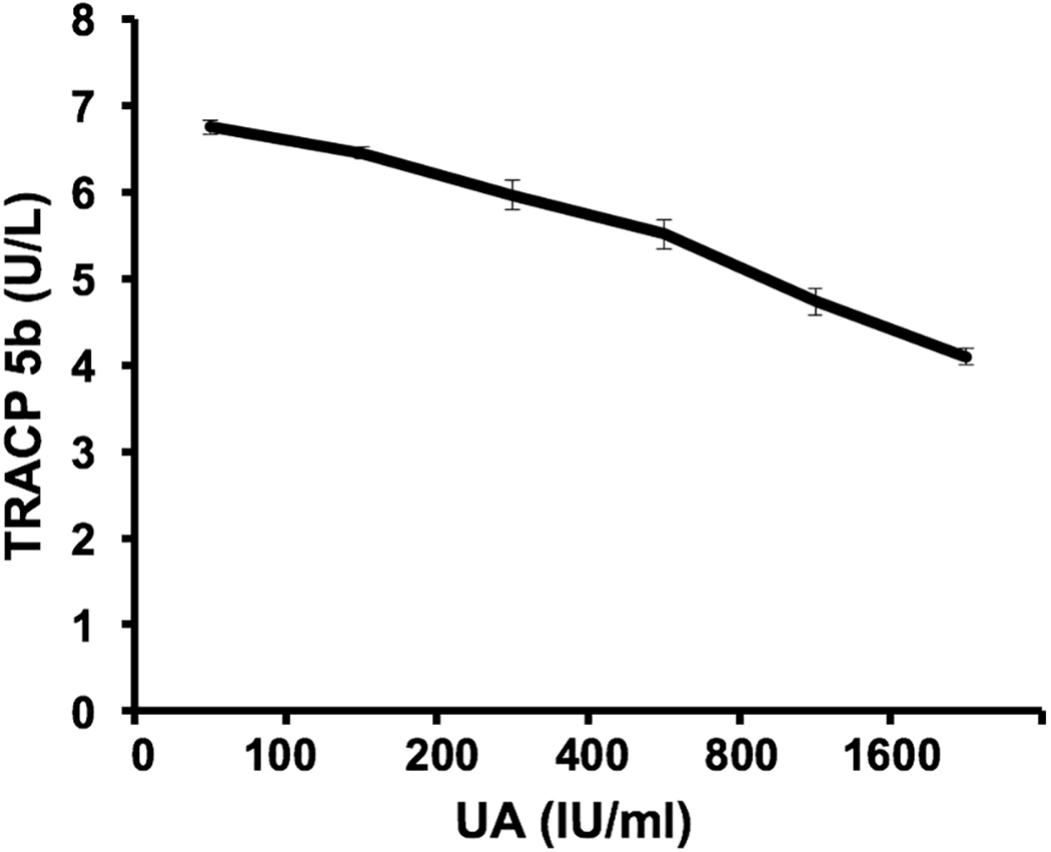
High concentrations of UA lead to decrease of TRACP 5b levels. A TRACP 5b standard sample was divided into 6 aliquots. Serial dilutions of UA (250, 500, 1000, 2000, 4000μM) were prepared from UA standard subject. The 5 aliquots were then spiked at 3:2 with each UA solution. This generated 5 different test samples with the same TRACP 5b level, whose final UA concentrations ranged from 100 to 1600 μM. An aliquot containing DDW instead of UA served as a blank. The average TRACP 5b concentration is marked with error bars representing standard deviations of three independent experiments.

**Fig 3:**
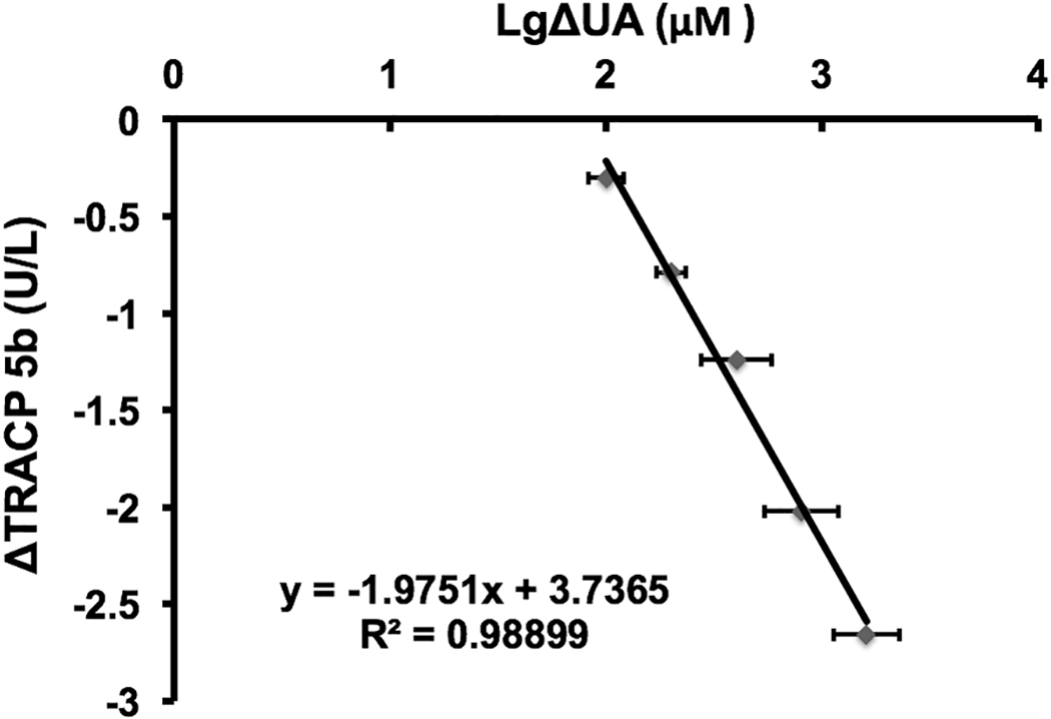
Derivation of a UA correction equation. The TRACP 5b concentrations of the six distinct test samples with the same TRACP 5b standard sample and final UA concentrations ranging from 0 to 1600 μM were measured. ΔTRACP 5b means difference of TRACP 5b concentrations measured between the samples of final UA concentrations ranging from 100 to 1600 μM and that of no UA. The average ΔTRACP 5b concentration is marked in figure with error bars representing standard deviations.

**Fig 4:**
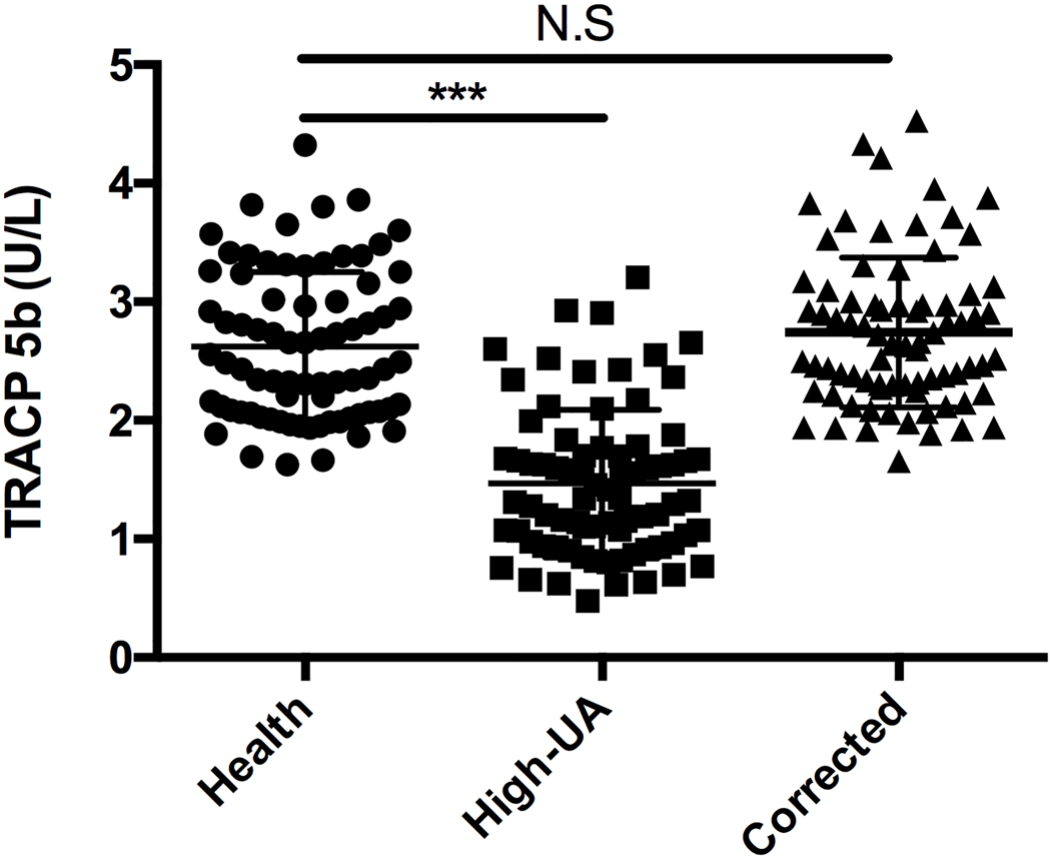
Correction of decreased TRACP 5b concentrations resulting from high-UA individuals compared to healthy subjects. Each high-UA individual's UA-corrected TRACP 5b concentration was calculated based on the UA-TRACP 5b correction equation. Increasing UA concentration means difference value between each high-UA individual's and mean value of healthy subjects’. Triple asterisk indicates p<0.0001. “N.S” indicates no significant difference.

TRACP 5b is frequently used in clinical practice as a tool for cancer patients with bone metastasis. However, recently, we observed that sixteen cancer patients bone metastasis diagnosed by bone scintigraphy showed no obviously increased TRACP 5b levels (TRACP 5b concentration: 3.99 ± 0.41 U/L). Meanwhile, these patients showed relatively higher serum UA concentrations (UA concentration: 398.4 ± 40.4 IU/ml). So we inspected that high UA concentrations were associated with decrease of TRACP 5b levels for cancer patients bone metastasis. To evaluate whether there was a correlation between high concentrations of UA and decrease of TRACP 5b levels, we randomly tested the TRACP 5b levels of 77 high-UA individuals (UA concentration: 463.5 ± 50.8 IU/ml) and 77 healthy subjects (UA concentration: 293.4 ± 56.9 IU/ml). The results showed that the average TRACP 5b levels of the high-UA individuals (1.47± 0.62 U/L) were significantly lower (t-test, p<0.0001) than that of the healthy subjects (2.62 ± 0.63 U/L) (Table 1) (Fig 1A–1C)(S1 File). These data indicated that high concentrations of UA lead to decrease of TRACP 5b levels is not an individual case that occurred in few cancer patients with bone metastasis but a universal phenomenon. We think there are at least two possible reasons for this. The first possible reason is that high concentrations of UA directly down-regulate expression of TRACP 5b at mRNA or protein level. The second possible reason is that high concentrations of UA interfere with TRACP 5b tests. As we know, UA could lower the value for glucose as determined by “GOD-Perid” method[32]. Here, we firstly checked if high concentrations of UA interfered with TRACP 5b tests. We conducted an interference experiment. As mentioned in the “Materials and Methods”, serial dilutions of UA were spiked into TRACP 5b standard samples. As a result, we observed dose-dependent decrease in TRACP 5b concentrations (Fig 2). After normalizing the measured TRACP 5b in each sample by dislodging the TRACP 5b level of the aliquot containing DDW instead of UA, then marking the change as a function of increment of UA, the least squares linear fit was set for ΔTRACP 5b (U/L) and marked as a function of UA (μM): ΔTRACP 5b (U/L) = -1.9751lgΔUA (μM) + 3.7365 with an R^2^ = 0.98899 (Fig 3). This implied 0.21 U/L false-decrease in TRACP 5b for each 100μM increase of UA concentration. Application of the UA correction resulted in a statistically non-significant difference in TRACP 5b values between the healthy subjects and high-UA individuals (p = 0.24)(Fig 4), which suggested that concentrations of UA were the key cause of differences in TRACP 5b level between the high-UA individuals and the healthy subjects. Therefore, we believed that the differences of TRACP 5b levels were mainly caused by a method-related systematic error. In addition, it was recently reported that higher UA level suppressed osteoclastogenesis, indicating that UA might lower TRACP 5b level [33]. We will further elucidate whether UA has direct effects in expression of TRACP 5b in our future work.

**Table 1:** Multiple parameters of serum samples and statistical analyses between groups.

Parameter	Study group	Control group	P value
**Number of patients (n)**	77	77	/
**Gender (male/female)**	61/16	61/16	/
**Age (years, mean ± SD)**	40.58 ± 12.60	42.24 ± 11.47	0.883
WBC (*10^**9**^**/L, mean ± SD)**	6.65 ± 1.20	6.62 ± 1.19	0.993
**NEU % (mean ± SD)**	57.41 ± 7.98	55.58 ± 9.29	0.895
**ALT (U/L, mean ± SD)**	26.81 ± 9.63	22.99 ± 7.17	0.637
**AST (U/L, mean ± SD)**	22.28 ± 4.99	21.37 ± 4.22	0.903
**AFP (ng/mL, mean ± SD)**	2.67 ±1.41	2.97±1.70	0.901
**CEA (ng/mL, mean ± SD)**	1.95±1.03	2.32±1.14	0.859
**Glu (mmol/L, mean ± SD)**	5.33 ± 0.39	5.19 ± 0.49	0.966
**BUN (mmol/L, mean ± SD)**	5.51 ± 0.99	5.31 ± 0.92	0.952
**CREA (μmol/L, mean ± SD)**	82.27 ± 14.44	70.68 ± 11.70	0.506
**UA ((μmol /ml, mean ± SD)**	463.50 ± 50.76	293.39 ± 56.89	0.009*
**TRACP 5b (U/L, mean ± SD)**	1.47 ± 0.62	2.62 ± 0.63	0.000*

WBC refers to white blood cells, NEU refers to neutrophils, ALT refers to alanine aminotransferase, AST refers to aspartate aminotransferase, AFP refers to alpha-fetoprotein, CEA refers to carcinoembryonic antigen, Glu refers to blood glucose, BUN refers to blood urea nitrogen, CREA refers to creatinine, UA refers to uric acid and TRACP 5b refers to tartrate-resistant acid. An independent sample t-test was employed (“*” indicates p<0.05).

## Conclusion

In this study, by analyzing the collected data and the result of an interference experiment in vitro, we illustrated that the patients with high concentrations of UA presented a false-negative decrease in TRACP 5b. Our results reminded physicians should fully consider interference of hyperuricemia, especially when TRACP 5b was used for early diagnosis of cancer patients with bone metastasis, evaluation of the aggressiveness of osteosarcoma or prediction of survival in prostate cancer and breast cancer with bone metastases.

## Supporting Information

S1 File The data used to make comparisons between 77 high UA and 77 healthy subjects. (PDF)

## References

[1] Johansson JE, Andrén O, Andersson SO, Dickman PW, Holmberg L, Magnuson A, et al Natural history of early, localized prostate cancer. JAMA. 2004;291: 2713–2719. 15187052 10.1001/jama.291.22.2713

[2] Zhang L, Li XS, Zhou LQ. Natural History of Small Renal Masses. Chin Med J. 2015;128: 1232–1237. 10.4103/0366-6999.156139 25947408 PMC4831552

[3] Wu YY, Janckila AJ, Ku CH, Yu CP, Yu JC, Lee SH, et al Serum tartrate-resistant acid phosphatase 5b activity as a prognostic marker of survival in breast cancer with bone metastasis. BMC Cancer. 2010;10: 158–159. 10.1186/1471-2407-10-158 20416078 PMC2873389

[4] Jung K, Lein M, Stephan C, Von Hosslin K, Semjonow A, Sinha P, et al Comparison of 10 serum bone turnover markers in prostate carcinoma patients with bone metastatic spread: Diagnostic and prognostic implications. Int J Cancer. 2004;111: 783–791. 15252851 10.1002/ijc.20314

[5] Terpos E, Kiagia M, Karapanagiotou EM, Charpidou A, Dilana KD, Nasothimiou E, et al The Clinical Significance of Serum Markers of Bone Turnover in NSCLC Patients: Surveillance, Management and Prognostic Implications. Anticancer Res. 2009; 29:1651–7. 19443381

[6] Chao TY, Yu JC, Ku CH, Chen MM, Lee SH, Janckila AJ, et al Tartrate-resistant acid phosphatase 5b is a useful serum marker for extensive bone metastasis in breast cancer patients. Clin Cancer Res. 2005;11: 544–550. 15701839

[7] Korpela J, Tiitinen SL, Hiekkanen H, Halleen JM, Selander KS, Väänänen HK, et al Serum TRACP 5b and ICTP as markers of bone metastases in breast cancer. Anticancer Res. 2006;26: 3127–3132. 16886645

[8] Recker R, Lappe J, Davies KM, Heaney R. Bone remodeling increases substantially in the years after menopause and remains increased in older osteoporosis patients. J Bone Miner Res. 2004;19: 1628–1633. 15355557 10.1359/JBMR.040710

[9] Bonjour JP, Benoit V, Rousseau B, Souberbielle JC. Consumption of Vitamin D-and Calcium-Fortified Soft White Cheese Lowers the Biochemical Marker of Bone Resorption TRAP 5b in Postmenopausal Women at Moderate Risk of Osteoporosis Fracture. Journal of Nutrition. 2012;142: 698–703. 10.3945/jn.111.153692 22357739

[10] Boutroy S, Van Rietbergen B, Sornay-Rendu E, Munoz F, Bouxsein ML, Delmas PD. Finite element analysis based on in vivo HR-pQCT images of the distal radius is associated with wrist fracture in postmenopausal women. J Bone Miner Res. 2008;23: 392–399. 17997712 10.1359/jbmr.071108

[11] McNeil BJ. Value of bone scanning in neoplastic disease. Semin Nucl Med; 1984;14: 277–286. 6387915 10.1016/s0001-2998(84)80003-3

[12] Ebert W, Muley T, Herb KP, Schmidt-Gayk H. Comparison of bone scintigraphy with bone markers in the diagnosis of bone metastasis in lung carcinoma patients. Anticancer Res. 2004;24: 3193–3201. 15510610

[13] Halleen JM, Alatalo SL, Janckila AJ, Woitge HW, Seibel MJ, Väänänen HK. Serum tartrate-resistant acid phosphatase 5b is a specific and sensitive marker of bone resorption. Clinical Chemistry. 2001;47: 597–600. 11238321

[14] Halleen JM, Alatalo SL, Suominen H, Cheng S, Janckila AJ, Väänänen HK. Tartrate-resistant acid phosphatase 5b: a novel serum marker of bone resorption. J Bone Miner Res. 2000;15: 1337–1345. 10893682 10.1359/jbmr.2000.15.7.1337

[15] Wu YY, Janckila AJ, Ku CH, Yu CP, Yu JC, Lee SH, et al Serum tartrate-resistant acid phosphatase 5b activity as a prognostic marker of survival in breast cancer with bone metastasis. BMC Cancer. 2010;10: 158 10.1186/1471-2407-10-158 20416078 PMC2873389

[16] Alatalo SL, Peng Z, Janckila AJ, Kaija H, Vihko P, Vaananen HK, et al A novel immunoassay for the determination of tartrate-resistant acid phosphatase 5b from rat serum. J Bone Miner Res. 2003;18: 134–139. 12510815 10.1359/jbmr.2003.18.1.134

[17] Terpos E, la Fuente de J, Szydlo R, Hatjiharissi E, Viniou N, Meletis J, et al Tartrate-resistant acid phosphatase isoform 5b: A novel serum marker for monitoring bone disease in multiple myeloma. Int J Cancer. 2003; 106: 455–457. 12845688 10.1002/ijc.11247

[18] Sarvari BKD, Sankara Mahadev D, Rupa S, Mastan SA. Detection of Bone Metastases in Breast Cancer (BC) Patients by Serum Tartrate-Resistant Acid Phosphatase 5b (TRACP 5b), a Bone Resorption Marker and Serum Alkaline Phosphatase (ALP), a Bone Formation Marker, in Lieu of Whole Body Skeletal Scintigraphy with Technetium99m MDP. Indian J Clin Biochem. 2015;30: 66–71. 10.1007/s12291-013-0399-8 25646043 PMC4310848

[19] Tang C, Liu Y, Qin H, Li X, Guo W, Li J, et al Clinical significance of serum BAP, TRACP 5b and ICTP as bone metabolic markers for bone metastasis screening in lung cancer patients. Clin Chim Acta. 2013;426: 102–107. 10.1016/j.cca.2013.09.011 24055775

[20] Chao TY, Wu YY, Janckila AJ. Tartrate-resistant acid phosphatase isoform 5b (TRACP 5b) as a serum maker for cancer with bone metastasis. Clin Chim Acta. 2010;411: 1553–1564. 10.1016/j.cca.2010.06.027 20599857

[21] Kamiya N, Suzuki H, Yano M, Endo T, Takano M, Komaru A, et al Implications of serum bone turnover markers in prostate cancer patients with bone metastasis. Urology. 2010;75: 1446–1451. 10.1016/j.urology.2009.11.049 20206975

[22] Janckila AJ, Yam LT. Biology and clinical significance of tartrate-resistant acid phosphatases: new perspectives on an old enzyme. Calcif Tissue Int. 2009;85: 465–483. 10.1007/s00223-009-9309-8 19915788

[23] Avnet S, Longhi A, Salerno M, Halleen JM, Perut F, Granchi D, et al Increased osteoclast activity is associated with aggressiveness of osteosarcoma. Int J Oncol. 2008;33: 1231–1238. 19020756

[24] Savarino L, Avnet S, Greco M, Giunti A, Baldini N. Potential role of tartrate-resistant acid phosphatase 5b (TRACP 5b) as a surrogate marker of late loosening in patients with total hip arthroplasty: a cohort study. J Orthop Res. 2010;28: 887–892. 10.1002/jor.21082 20063383

[25] Salminen EK, Kallioinen MJ, Ala-Houhala MA, Vihinen PP, Tiitinen SL, et al Survival markers related to bone metastases in prostate cancer. Anticancer Res. 2006;26: 4879–4884. 17214355

[26] Chung YC, Ku CH, Chao TY, Yu JC, Chen MM, Lee SH. Tartrate-Resistant Acid Phosphatase 5b Activity Is a Useful Bone Marker for Monitoring Bone Metastases in Breast Cancer Patients after Treatment. Cancer Epidemiology Biomarkers & Prevention. 2006;15: 424–428. 10.1158/1055-9965.EPI-04-0842 16537696

[27] Ghaemi-Oskouie F, Shi Y. The role of uric acid as an endogenous danger signal in immunity and inflammation. Curr Rheumatol Rep. 2011;13: 160–166. 10.1007/s11926-011-0162-1 21234729 PMC3093438

[28] Giovannucci E. Metabolic syndrome, hyperinsulinemia, and colon cancer: a review. Am J Clin Nutr. 2007;86: s836–s842. 18265477 10.1093/ajcn/86.3.836S

[29] Rose DP, Haffner SM, Baillargeon J. Adiposity, the metabolic syndrome, and breast cancer in African-American and white American women. Endocr Rev. 2007;28: 763–777. 17981890 10.1210/er.2006-0019

[30] Hammarsten J, Damber JE, Peeker R, Mellström D, Högstedt B. A higher prediagnostic insulin level is a prospective risk factor for incident prostate cancer. Cancer Epidemiol. 2010;34: 574–579. 10.1016/j.canep.2010.06.014 20702155

[31] Fini MA, Elias A, Johnson RJ, Wright RM. Contribution of uric acid to cancer risk, recurrence, and mortality. Clin Transl Med. 2012;1: 16 10.1186/2001-1326-1-16 23369448 PMC3560981

[32] Chinh NH. Mechanism of interference by uric acid in the glucose oxidase-peroxidase method for serum glucose. Clinical Chemistry. 1974;20: 499–501. 4818206

[33] Ahn SH, Lee SH, Kim BJ, Lim KH, Bae SJ, Kim EH, et al Higher serum uric acid is associated with higher bone mass, lower bone turnover, and lower prevalence of vertebral fracture in healthy postmenopausal women. Osteoporos Int. 2013;24: 2961–2970. 10.1007/s00198-013-2377-7 23644878

