# Supplementary material for: High Uric Acid (UA) Negatively Affects Serum Tartrate-Resistant Acid Phosphatase 5b (TRACP 5b) Immunoassay
Source: PLoS One. 2016 Jan 22;11(1):e0147554. doi: 10.1371/journal.pone.0147554 (PMC4723035; doi:10.1371/journal.pone.0147554)
Supplement: S1 File — (PDF) [file pone.0147554.s001.pdf]

Sample No.	Patient No.	Gender	Age	UA	TRACP 5b	WBC	NEU%	ALT	AST	GLU	BUN	Cr	AFP	CEA
1	263928	M	17	432	2.366	5.8	58.7	20.1	19.3	5.6	5.09	83	1.4	1.9
2	257555	M	23	490.7	2.602	9.16	61.9	49.7	24.5	4.94	4.92	85.6	1.2	2.1
3	256928	M	25	524	2.119	6.9	57.6	39.8	25.9	5.63	5.29	84	1.9	3.8
4	256442	M	25	489	1.594	6.35	45	18.8	15.1	5.26	4.99	84.5	2.3	3
5	263127	M	25	453	1.78	8	60.4	21.5	19.8	4.72	7.8	55.7	3.4	1.3
6	260970	M	25	460	0.939	8.12	60.5	32.7	19.6	5.55	5.45	84.6	1.4	3.1
7	256533	M	26	522	1.161	7	64.6	25.8	18.1	5.12	5.52	85.7	1.3	2.5
8	261854	M	26	464	1.555	7.6	64.3	47.1	21.9	4.82	5.27	74.2	1.6	0.6
9	257641	M	27	545	2.551	7.4	53.3	40.7	21.3	4.57	5.74	83.6	2	1.2
10	263128	M	27	439	1.338	6.9	57.1	49.1	25	4.38	7.23	73.2	2.2	3.1
11	252899	M	27	582	0.818	6.27	50.1	28.2	22.2	4.92	5.73	75.9	2	3.1
12	259099	M	28	450.7	1.837	7	58.8	20.9	25.1	5.39	5.08	81.7	1.8	1.6
13	258259	M	29	561	2.427	6.3	47.5	25.7	36.1	5.78	6.49	96.3	0.8	1.8
14	261113	M	29	431	0.614	7.7	63	22.1	17.2	5.49	6.87	70.7	2.3	1.1
15	256571	M	30	476	1.672	6.4	48	29.8	24.6	5.83	5.64	98.9	2	2.9
16	259029	M	30	442	1.698	5.8	50.8	36.4	25.4	5.06	5.45	93	4.4	2.6
17	261488	M	30	462	1.625	6.05	60	15.6	19.7	4.96	5.84	100	2.4	1.6
18	257549	M	31	473.5	2.344	6.69	50.5	26.8	20.8	5.31	4.81	78.2	1.6	1.6
19	258990	M	31	435	1.542	7.11	66.9	23.2	18.4	5.42	5.88	83.1	2.2	2.7
20	261105	M	31	535	1.625	7	68.5	46	22.6	5.8	5.78	76.7	2	2.2
21	261528	M	31	473.2	1.204	8	55.7	45.7	32.5	5.63	4.5	82.1	1.8	1.4
22	250251	M	32	441	0.758	6.8	64.9	19.2	20.3	5.51	3.81	73.6	1.1	3.5
23	256909	M	33	460	1.322	4.6	40.4	18.1	12.7	5.57	5.9	98.5	2.6	1.4
24	257888	M	33	482	2.409	4.2	51.2	16.4	11.1	5.29	6.45	86.8	2.1	0.7
25	252783	M	34	498.1	2.657	6.6	62.7	21.3	21.6	5.13	6.6	82.2	2	1.6
26	3787202	M	35	523	0.696	6.27	72.7	31.8	23.7	5.7	5.16	81.9	4.2	2.7
27	261187	M	35	517	1.139	6.55	47.2	25.8	18.4	5.28	5.28	82.6	3.4	2.5
28	251109	M	36	454	1.458	6.11	55.4	17.5	20.6	5.26	4.68	83.2	4.4	2.7
29	256497	M	36	462.1	1.659	6.64	56.7	15.7	13.5	5.27	4.93	70.8	4.1	0.5
30	261491	M	36	492.8	1.07	4.6	63.5	24.1	23.9	4.91	5.79	116.2	3.4	1.8
31	255860	M	38	438	0.965	5.54	62.2	37.7	27.2	5.6	4.54	82.3	2.4	4.5
32	263077	M	38	526	1.135	5.76	52.3	23.4	20.9	4.5	5.85	102.6	2.2	3.2
33	249644	M	41	430	1.072	5.06	52.7	21.9	25.4	5.6	5.98	88.7	1.8	3.4
34	241571	M	41	523	1.276	7.8	67	22.9	24.4	4.86	6.31	77	3	1.2
35	256478	M	41	470.7	1.189	5.73	63	28.3	19.5	5.52	6.56	91.4	6	1.8
36	262640	M	41	466.8	2.171	4.3	56.6	23	30	4.51	6.6	85.5	5.2	0.6
37	257509	M	42	453.2	0.636	8.14	67.6	40.2	22.3	4.67	5.27	84.8	2.5	3.5
38	236595	M	45	438	1.413	6.2	50.7	14.4	19.7	5.8	3.7	92.1	4.2	2.4
39	260168	M	46	432.9	1.343	7.02	51.9	32.4	22.7	5.52	4.65	69	4.7	2.8
40	249609	M	47	533	0.478	5.7	51.3	31.9	25.6	5.32	5.98	92.6	3	0.8
41	249611	M	47	501	1.032	6.42	63.9	31.4	23.8	5.8	5.02	95.5	5	2.3
42	244490	M	47	445	1.677	4.7	40.6	28.4	19.6	5.63	5.92	80.3	2.5	2.3
43	262530	M	47	440.3	2.908	6.3	49	20.9	24.5	5.66	5.4	87	2.8	4.2
44	249585	M	49	522	0.931	6.8	59.3	43	37.5	5.76	5.28	121.2	2.8	1.9

45	257645	M	49	455	1.994	5.91	44.1	22.7	18.5	5.81	5.1	92	3.7	1.7
46	13683	M	49	511	1.703	9.25	63.3	25.2	22.1	5.02	6.27	129.3	2.5	1.2
47	260188	M	49	548.1	0.909	6.9	60.3	35.7	25.8	5.34	5.2	95.3	1.9	1.3
48	252891	M	50	432.8	3.208	7.3	46.4	10.5	22.9	5.55	7.44	84.4	1.1	1.5
49	249569	M	51	501	0.903	5.16	57.6	26.1	26.9	5.42	3.96	88.8	4.9	0.9
50	249571	M	51	441	1.072	7.73	66.7	32.9	18.7	5.46	4.38	89	2.5	0.9
51	555078	M	51	513	1.105	4.72	59.1	30.4	22.2	5.18	7.48	108	2.4	1.4
52	241949	M	51	502	0.931	9.11	56.7	48.7	30.2	5.27	5.2	77.9	5.8	3.6
53	263674	M	53	488	1.633	5.3	41.2	31.9	30.9	4.98	6.72	80	3.4	2
54	256468	M	54	513	1.295	5.9	58.1	17.2	23.47	4.98	5.88	86	2.8	0.6
55	253462	M	57	450	1.152	6.17	55.1	27.1	24.8	5.98	5.54	88.6	4.6	2.7
56	258469	M	58	482.4	1.58	4.7	47.7	38.4	21.9	5.64	6.99	93.6		1.1
57	249504	M	62	454.2	0.803	6.4	67.3	17.6	25.3	6	4.51	78.4	4.2	2.2
58	263016	M	62	508	1.312	5.6	48.9	19.7	20	4.8	6.72	84.8	3.6	4.4
59	249489	M	64	433	0.623	9.24	68.4	29.6	24.3	5.81	5.81	91.5	1	2
60	249791	M	66	466	0.657	5.93	66.4	21.3	19.3	5.55	5.23	84.3	8.6	4.3
61	256262	M	66	430	1.077	7.65	60.2	28.8	18	5.27	5.53	63.1	1.8	1.4
62	260865	F	25	361	0.77	7.8	55.4	27.6	22.1	4.64	5.05	57.2	1.2	1.1
63	260503	F	28	401.3	1.599	8.5	59.2	17.1	18.1	5.16	5.78	67.8	1.8	1.4
64	263172	F	30	387.7	0.978	5.16	45.3	21.4	24.9	5.43	4.99	64.1	1.9	1
65	250273	F	30	376.4	1.208	8.08	65.3	29.4	21.2	4.73	4.27	57.1	1.4	1.4
66	256247	F	31	386.2	0.848	7.74	48.9	22.2	23.1	5.5	4.31	58.4	3.6	0.6
67	256637	F	32	403	1.552	7	61.2	7.9	11	5.76	4.24	85.8	2.5	0.2
68	256846	F	35	499	1.879	7.8	42.7	31.2	28.7	5.27	4.74	86.8	2	0.9
69	257988	F	35	370.7	2.096	7.7	64.2	13.3	14.3	5.42	3.31	58.3	0.9	0.7
70	257133	F	46	434	1.658	8.29	51.8	19	18.5	5.41	3.5	60.4	3.1	2
71	258754	F	46	380	1.104	8.3	69.7	26.7	15.8	5.91	5.33	67.5	1.3	2.8
72	257624	F	50	384.6	1.769	7.5	56.5	37.8	32.3	4.67	3.47	57.9	1.4	1.5
73	261232	F	52	372.4	0.87	6.4	64.2	11.3	21.4	5.38	5.56	62.5	1	2.3
74	258486	F	57	373	2.524	6.5	58.2	17.4	16.5	5.74	6.81	56.8	1.2	1.3
75	263625	F	60	413.2	1.616	5.7	70.1	19.1	22.2	5.52	5.96	73.3	2.1	1
76	3157995	F	66	542.7	0.814	5.33	69.5	21.8	26	5.58	7.3	82.1	1.8	1.7
77	261779	F	66	385.8	2.929	6.6	65.4	22.1	20.7	5.7	7.18	67.4	3.8	0.3
Average			40.58	463.5	1.467221	6.6592	57.417	26.812	22.29	5.3342	5.5168	82.271	2.6737	1.9597
SD			12.6	50.7656	0.621403	1.2061	7.9806	9.6375	4.994	0.3925	0.9918	14.449	1.4118	1.038

Sample No.	Ctrl No.	Gender	Age	UA	TRACP 5b	WBC	NEU%	ALT	AST	GLU	BUN	Cr	AFP	CEA
1	262693	M	33	261.1	1.664	5.83	56.6	17.6	15.7	5.46	7.36	82.6	1.5	4.1
2	262629	M	35	369.6	2.076	5.2	34	26.3	23.9	5.21	5.32	82.7	1.9	2.4
3	258383	M	38	275	2.731	7.8	60.7	33.2	28.6	5.23	6.06	93.5	2.1	2
4	262701	M	43	284.3	1.937	7.5	62.3	24	21.2	5.4	5.47	88.9	1.9	1.3
5	262548	M	43	240.7	2.306	6.36	61.7	20.1	21.3	5.15	5.47	83.9	3.5	3.6
6	257695	M	47	298	3.316	8.87	54.9	16	17.2	5.64	5.41	86.1	2.2	1.9
7	262957	M	51	203.2	2.431	4.8	38.8	19.1	20.7	5.57	4.67	102.3	4.6	2.9
8	263683	M	51	266	2.028	5.05	50.1	16.9	17.3	5.41	5.19	65.6	1.3	0.7
9	259944	M	54	261.4	2.338	6.6	54.5	16.6	26.2	5.34	5.69	80.9	10.2	4.6
10	259949	M	54	305.3	2.201	7.1	66.6	19	16.3	4.95	6.78	74.3	2.7	1.4
11	262692	M	44	363	3.328	6.1	42.8	31.2	22.9	5.74	3.25	75.7	2.1	1.7
12	262919	M	57	320	2.732	5.7	66.2	27.4	29.2	5.14	4.98	69.8	2.9	2.3
13	262700	M	35	269	3.02	8	55.3	25.8	28.4	4.85	6.3	71.2	2.9	5.1
14	262962	M	54	280	2.311	8	68.1	17.2	24.8	5.13	5.61	68	1.4	2.7
15	262922	M	59	367	2.211	8.77	51	25.2	20.6	6.05	4.53	71.4	1.5	3.9
16	258546	M	50	326	3.415	4.99	47.5	25.5	25	5.2	5.69	77.9	2.5	2.3
17	262990	M	54	282	2.117	7.18	65.5	23.3	17.7	5.21	4.73	60.3	1.3	3.5
18	262802	M	40	346	2.556	6.9	55.9	26.9	23.5	2.29	5.17	79.8	3	1.2
19	264415	M	44	356	3.861	7.12	60.4	20.2	18.9	5.63	4.35	86.6	4.4	1.7
20	264419	M	45	363	3.817	6	63.6	23	26.6	5.48	6.1	67.4	1.5	1.4
21	6988	M	58	280	2.688	6.46	56.1	17	21.8	5.17	4.29	73.2	4.7	2.4
22	262812	M	25	312	2.431	4.82	50.1	33.3	21.1	5.22	4.42	66.9	2.5	3.3
23	262651	M	41	294	2.33	5.2	48.5	16.5	19.3	4.93	4.33	77.4	3.8	2.9
24	263896	M	46	341	2.807	3.14	43.3	25.5	28.9	4.75	5.19	66.9	3.5	1.5
25	262601	M	49	288	3.604	7.13	63.2	15.1	18.5	4.87	4.92	84	5	4.6
26	262105	M	31	368	3.302	9.1	71.2	35.7	22.9	5.17	4.01	74.9	1.7	0.9
27	262595	M	41	288	2.656	7.02	60.3	23.5	18.8	5.44	6.24	73.6	2.9	2.5
28	262141	M	43	285	2.067	6.9	62.7	19	20.3	4.69	5.21	73.7	6.3	1.7
29	262605	M	49	254	2.136	8.2	74.5	20.7	17.4	5.54	7.35	67.1	5.8	3.1
30	262778	M	22	355	2.487	6.42	56.4	24.1	21.5	5.38	3.24	71.4	1.6	2.1
31	262943	M	59	406	2.01	5.9	58	38	28.5	5.88	5.89	72.6	4.7	3
32	262921	M	56	263	1.91	7.33	48.4	36.9	24.9	5.31	7.02	73	2.9	1.8
33	262709	M	31	219	3.24	7.06	45.9	41.3	38.8	6.06	4.84	56.4	4.1	4.8
34	262795	M	48	282	3.572	5.75	65.2	30.6	26.1	5.66	5.85	64.8	2.7	1.8
35	262901	M	38	398	3.334	7.14	50.4	34.2	20.3	5.54	5.93	68.3	3	1.8
36	262845	M	46	362	2.06	5.9	49.8	30.9	24.7	5.63	5.85	83.4	3.3	1.4
37	262456	M	50	371	3.384	6.77	64	23.6	22.9	5.32	5.43	65.3	2.9	1.2
38	262838	M	29	316	1.954	5.18	51.5	15.3	17.5	4.96	3.63	77.7	2.2	2.9
39	262975	M	50	357	2.656	6.66	61.2	20.9	16.6	5.88	5.7	65	2.4	2.6
40	262815	M	46	225	2.763	6.72	46.4	17.9	19.7	5.24	5.24	66.9	1.6	1
41	262948	M	50	290	1.885	5.69	48.3	20.2	20.4	5.31	6.12	84	1.5	2.8
42	262621	M	41	323	2.826	6.4	48.5	36.3	26.1	4.91	5.99	67.7	2.9	4.1
43	263687	M	27	318	2.945	7.08	54.6	38.7	26.2	4.72	5.96	72.3	1.9	1.4
44	262744	M	27	318	2.343	5.66	41.5	20.8	17.6	5.11	5.76	78.4	2.3	1.5

45	263557	M	59	377	3.252	6.6	58.7	25.8	17.8	5.14	6.02	82.6	2.1	1.3
46	262773	M	23	303	2.324	6	46.9	29.1	29.6	4.8	4.37	79.6	2.1	4.1
47	262786	M	22	312	1.866	5.9	54.2	22.2	17.7	4.87	5.79	63.7	2.4	3.6
48	261358	M	53	325	1.954	6	50.3	19.8	16.5	4.69	5.77	89.3	1.6	1.9
49	262842	M	40	394	2.324	7.34	63.6	36.2	18.3	5.77	5.77	74.8	3	1.2
50	262793	M	36	281	2.161	5.69	54.8	14.5	18.9	5.17	5.07	69.2	2.6	2.2
51	262478	M	36	273	2.086	4.3	50.6	22.7	24.2	5	5.78	86.8	3.6	4.1
52	262706	M	25	286	2.098	8	83.7	30.4	21.5	5.61	5.18	77.8	1.6	1.3
53	261346	M	46	365	2.493	6.65	47.1	20.2	17.4	4.95	5.64	66	2.8	2.4
54	262986	M	51	229	3.391	6.8	42.5	25.6	18.9	4.94	5.24	68.5	5.1	4.2
55	262854	M	42	354	2.769	7.9	56.1	20.9	17.8	5.4	6.9	74.7	4.6	3.4
56	262729	M	36	347	3.001	6.66	54.5	24.5	22.2	5.04	4.57	60.9	4.4	1.4
57	262261	M	44	273	2.048	6.26	55.4	31	23.5	5.18	5.32	60.9	0.9	4.3
58	264386	M	40	254	2.017	8.5	58	27	19.2	4.95	6.14	79.4	3.6	1.2
59	286658	M	27	276.7	2.871	5.86	62.1	14.2	21.9	4.83	6.62	65.3	6.3	1.4
60	287271	M	28	339.3	2.819	7.4	56.4	17.6	25.7	5.66	4.93	83	2.4	2.5
61	287532	M	22	304.3	2.965	7.01	64.2	23	19.9	4.98	4.39	75.9	2.6	3.5
62	248402	F	32	274.7	1.626	8.16	66.6	20.4	18.3	4.74	7.17	60.9	1.2	1.6
63	264287	F	39	237	3.485	7.9	73.6	13.8	15.2	5.83	3.69	56.1	2	4
64	262575	F	47	328	1.989	7.3	50	15.4	21.8	5.03	5.7	62.9	2.1	1.2
65	264295	F	48	192	1.985	6.2	54	13.2	21.1	5.58	5.16	55.6	0.6	1.7
66	259950	F	52	117.8	1.695	5.4	49.2	21.8	20.2	5.2	4.38	58.8	3	1.8
67	260386	F	52	271.2	3.155	4.46	53.8	18.5	22.7	4.77	5.43	69.6	2.3	1.4
68	259945	F	64	254	3.653	7.8	61.8	16.37	22.8	5.39	5.64	48.2	9.6	2.3
69	264424	F	63	172	4.325	6.77	61.2	9.2	17.6	5.23	5.27	46.5	4.2	1.6
70	262506	F	27	180	3.804	4.86	52	16.2	22.1	4.5	3.2	46.3	2.3	3.4
71	261131	F	30	274	1.991	7.99	62.3	20.3	18.4	5.21	4.47	57.4	1.2	0.7
72	260861	F	29	265	2.355	6.3	56.9	13.1	18.9	4.86	5.56	64.7	3.4	2.1
73	264388	F	26	222	3.39	8.52	59.5	9.2	14.1	5.41	5.05	56.6	1.5	0.9
74	264373	F	31	212	2.067	5.21	37.4	24.2	18.5	4.79	4.31	48.7	4.6	0.9
75	264353	F	57	222	1.966	7	31	27.2	21.1	5.4	4.17	44.4	3.7	1
76	264305	F	66	280	3.259	6.8	50.4	17.2	20.4	5.99	6.03	67.1	3	1.7
77	262591	F	26	247	2.92	8.94	64.8	19.6	15.2	4.5	4.17	46.4	0.9	0.7
Average			42.25	293.397	2.62194805	6.6231	55.586	22.998	21.38	5.197	5.3174	70.681	2.9727	2.3221
SD			11.47	56.8907	0.6288973	1.1907	9.2968	7.1715	4.222	0.495	0.92025	11.701	1.7006	1.1492
